# Characteristics and Outcomes of Psoas Abscess: Experience From a Tertiary Care Center in North India

**DOI:** 10.7759/cureus.21358

**Published:** 2022-01-18

**Authors:** Ajay Thakral, Divya Prasad, Sachin Katyal, Arvind Kumar

**Affiliations:** 1 Department of General Surgery, Hamdard Institute of Medical Sciences and Research, New Delhi, IND; 2 Department of Orthopaedics, Hamdard Institute of Medical Sciences and Research, New Delhi, IND

**Keywords:** tuberculosis, psoas abscess, outcomes, north india, infection

## Abstract

Background

Psoas abscess (PA) is an uncommon disease involving infection of the psoas muscle with abscess formation. The evidence concerning clinical and diagnostic characteristics of PA and its outcomes is limited. The literature is heterogenous, with varying presentations and outcomes in different regions worldwide. We present a retrospective analysis of the clinical, radiological, and laboratory characteristics of PA, its management, and outcomes from a tertiary care center in North India.

Methodology

We reviewed the clinical records of confirmed cases of PA treated in our institute from January 2016 to December 2020 with a minimum follow-up of one year. Further, we performed a descriptive analysis of demographic characteristics, clinical features, laboratory parameters, radiological investigations, the basis of diagnosis confirmation, causative microorganisms, definitive management, treatment outcomes, and complications.

Results

We reviewed 33 cases with a mean age of 29.9 ± 16.8 years. Overall, 48.4% of PAs were right-sided, and 24.2% were bilateral. Abdominal discomfort was the most common presenting symptom. Blood laboratory parameters were mostly within the near-normal range except for the elevated erythrocyte sedimentation rate, C-reactive protein, and neutrophil-to-lymphocyte ratio. Ultrasonography was the most commonly performed radiological investigation and was the basis of diagnosis confirmation. *Mycobacterium tuberculosis* was the most common causative microorganism. Most patients required percutaneous drainage, and around one-fourth required open drainage. All patients had symptomatic as well as radiological improvement and no major complications.

Conclusions

Tuberculosis is the most prevalent cause of PA in the North Indian population. Most patients respond well to the less invasive treatment with percutaneous therapeutic drainage and antitubercular drugs, with few patients requiring open drainage. However, tissue diagnosis may remain inconclusive in a few patients, and antitubercular treatment may need to be initiated based on the clinicoradiological evaluation. Nevertheless, the rate of complications is low, with nil mortality probably related to the mild-to-moderate disease course of tuberculosis.

## Introduction

Psoas abscess (PA) is a rare condition in which the psoas muscle, with or without iliacus muscle involvement, gets infected, resulting in abscess formation [1]. The source of infection can be hematogenous due to this muscle’s rich vascularity, resulting in a primary PA or secondary due to the infection of adjacent organs, lymph nodes, or near vertebral bodies [2]. Besides this, several risk factors can contribute to PA formation, including, but not limited to, immunocompromised status, pre-existing regional or systemic infections, and local iatrogenic or traumatic injuries [3]. The etiology of PA is most commonly related to *Mycobacterium tuberculosis* infection. However, with the recent decline in tuberculosis, genitourinary and gastrointestinal infections have been frequently implicated in PA formation, especially in immunocompromised patients [4]. The classical presentation includes fever, back pain, and limp. However, these findings may not always be present [5]. Due to the rare nature of this disorder, the evidence concerning its etiology, natural history, ethnic variations, and outcomes is limited. Most evidence comes from small case series and reports [3-7]. In addition, not all surgeons are familiar with PA management, which can often be challenging.

This study retrospectively analyzed the clinical, radiological, and laboratory characteristics, management, and outcomes of PA in a tertiary care center in North India.

## Materials and methods

We retrospectively reviewed the clinical records of radiologically confirmed adult cases of PA that were treated in our institute from January 2016 to December 2020. We only considered cases with at least one year of follow-up. Patients who left the treatment at any stage were excluded. The data reviewed were grouped under the following heads:

(a) Demographic characteristics: The demographic characteristic of the patients, including age, gender, laterality, and region of origin, were noted.

(b) Clinical features: The clinical records were screened for classical symptoms of PA, namely, fever, back pain, and limp. Additional symptoms were also noted on an individual basis. In addition to these, we recorded the duration of symptoms, other medical or surgical conditions being treated, and any substance abuse, including alcohol intake and smoking.

(c) Laboratory parameters: Among laboratory parameters, we measured hemoglobin, total leucocyte count, neutrophil-to-lymphocyte ratio, erythrocyte sedimentation rate (ESR), C-reactive protein (CRP), serum albumin and globulin levels, liver function tests (serum bilirubin, serum glutamic oxaloacetic transaminase (SGOT), serum glutamic pyruvic transaminase (SGPT), and alkaline phosphatase (ALP)), and kidney function tests (serum urea and creatinine).

(d) Radiological investigations: We noted the type of radiological investigation(s) ordered, their main findings, and the need for higher radiological investigations. In addition, we noted the details regarding the extent of the abscess based on the reported findings.

(e) Confirmation of diagnosis: The basis of confirmation of the diagnosis of PA was noted in each case.

(f) Causative microorganism: The abscess culture reports were assessed, and the positive findings were noted.

(g) Definitive management: The type of management, namely, medical and surgical, was noted. The main surgical indications were noted for each reviewed case record.

(h) Treatment outcomes: The outcomes of the PA were graded as healed, improving, non-improving, and recurrence. The specific reason(s) for grading the outcomes among these groups was noted from the available records. In addition, we noted the further plan of management for non-improving cases.

(i) Complications: Any complications related to the disease or its management during the study period were noted.

We performed a descriptive analysis for the studied parameters retrieved from the clinical records. The continuous variables were reported as mean ± standard deviation (SD) with their interquartile ranges. The categorical variables were expressed as proportions.

## Results

Demographics

We reviewed 33 cases of PA meeting the eligibility criteria, including 20 (60%) female and 13 (40%) male patients. The mean age of the patients was 29.9 ± 16.8 years (range: 5-65 years). Overall, 48.4% of PAs were right-sided, and 24.2% were bilateral. All patients belonged to the North Indian region.

Clinical features

The clinical records suggested that only 51.51% of patients had a fever (all low grade), 36.36% of patients had low back pain, and only 15.15% had a limp or difficulty walking. Overall, 90.9% of patients complained of abdominal discomfort. No other major symptoms were observed among any of the reviewed patients. In total, 6% of patients were diabetic and hypertensive, 8% had a chronic respiratory illness, and 6% had osteoporosis. Among the concurrent infection-related symptoms and concomitant illnesses, additional infectious foci were observed to be Pott’s spine (53%), disseminated tuberculosis (3%), and pulmonary tuberculosis (3%). The mean duration of symptoms of the patients was 12.32 days (range: 2-52 days). Overall, 24.24% of patients had a history of chronic smoking, and 18% had a history of chronic alcohol intake.

Laboratory parameters

The blood investigations revealed elevated ESR, CRP, and neutrophil-to-lymphocyte ratio. The remaining blood parameters were near-normal or slightly altered (Table 1).

**Table 1:** Laboratory parameters of psoas abscess cases reviewed in this study. SD: standard deviation; SGOT: serum glutamic oxaloacetic transaminase; SGPT: serum glutamic pyruvic transaminase; ALP: alkaline phosphatase

Laboratory parameters	Mean ± SD	Interquartile ranges
Hemoglobin (g/dL)	10.3 ± 1.4	9.5–10.8
Total leucocyte count (count/mm^3^)	12.1 ± 7.3	7.4–12.6
Neutrophil-to-lymphocyte ratio	5.3 ± 4.3	2.2–6.8
Erythrocyte sedimentation rate (mm/hour)	83.5 ± 38.3	45–120
C-reactive protein (mg/dL)	21.4 ± 12.82	3.2–31.2
Serum albumin (g/dL)	3.3 ± 0.6	2.9–3.4
Serum globulin (g/dL)	4.2 ± 0.8	3.7–4.6
Serum bilirubin (mg/dL)	0.6 ± 0.6	0.3–0.8
SGOT (U/L)	43.5 ± 48.3	22–49.3
SGPT (U/L)	48.1 ± 52.3	17–53.3
ALP (U/L)	205.81 ± 180.37	113–221
Serum urea (mg/dL)	26.23 ± 13.89	18–31.3
Serum creatinine (mg/dL)	0.7 ± 0.3	0.5–0.9

Radiological investigations

Ultrasonography (USG) was performed in all of the reviewed cases and confirmed the diagnosis of PA in all included cases. Additional radiological investigations included magnetic resonance imaging (MRI) and computed tomography (CT). In total, 12 patients underwent MRI for associated back pain and clinical findings suggestive of spine disease. The diagnosis of Pott’s spine was made after MRI findings suggested vertebral involvement. In total, 11 patients underwent CT. In this series, the indications for CT were surgical planning for patients planned for open drainage, failed USG-guided percutaneous drainage, and recurrence of the abscess. The mean PA volume was 266.9 ± 382.9 (range: 20-1,500) mL. No associated abdominal visceral abnormalities were detected on USG and higher investigations.

Causative microorganisms

The abscess collection was either aspirated using a wide-bore needle through the prominent abscess zone, collected from the pigtail catheter inserted in the abscess cavity, or collected from operative drainage of the abscess. Bacteriological culture and cartridge-based nucleic acid amplification test (CBNAAT) reports were available for the abscess aspirate of the reviewed cases. *Staphylococcus aureus* was isolated in four (12.1%) patients, with one methicillin-resistant *Staphylococcus aureus* isolate, *Escherichia coli* was isolated in one (3%) of the patients, and two (6%) cases were positive for *Acinetobacter baumannii*. In total, 13 (39.4%) patients had positive CBNAAT reports suggestive of tuberculous infection. Two (6%) patients were positive for both bacterial culture and CBNAAT. No organism could be isolated in six (18.2%) cases. However, microscopic examination of the aspirates among these cases suggested fields full of neutrophils. In addition, nine (27.3%) cases had abscess volumes less than 100 mL, which were too deep to be aspirated. Based on the clinical evaluation and radiological findings, these patients were assumed to be tubercular infections.

Definitive management

Antitubercular treatment was administered to all reviewed cases based upon the clinical features of tubercular infection, irrespective of bacterial culture and CBNAAT reports. Additional antibiotics according to the sensitivity were administered in culture-positive collections. No multidrug-resistant tubercular infection was encountered. In addition, 15 (45.4%) patients underwent USG-guided pigtail catheter insertion for abscess drainage, nine (27.3%) underwent open surgical drainage of the abscess, and the remaining nine (27.3%) underwent medical management only. The nine patients who underwent medical management alone had clinicoradiological features of tubercular infection and were kept on antitubercular treatment only. The factors involving open drainage were more than two failed attempts of pigtail catheterization, multiloculated cavity upon radiological evaluation, and evidence of thick organized pus non-amenable for percutaneous drainage. It was based on surgeons’ preference when the collection was more than 1 L in two cases.

Treatment outcomes and complications

All patients showed improvement after initiating antitubercular drugs and their combination with bacterial-sensitive antibiotics in bacterial culture-positive cases. The pigtail catheters were removed when drainage through them had stopped and when USG evaluation revealed a progressive reduction in abscess volume of less than 100 mL. The mean duration of drain insertion was 16.4 (range: 5-36) days. The same criteria were utilized for open drainage-associated drains. All reviewed patients had completed the antitubercular treatment and had no clinical and USG-based signs of residual collection. The improvement was labeled based on symptomatic improvement, swelling regression, and drain collection reduction. Two patients were readmitted after discharge due to blockage of pigtail catheter within two weeks of insertion. The pigtail catheters were revised after USG confirmation of abscess persistence in both patients. One patient had abscess recurrence after three months of initial resolution. However, records suggested non-compliance to antitubercular drugs. The patient underwent another USG-guided catheter insertion, and antitubercular treatment was restarted. The repeat CBNAAT revealed no rifampicin resistance. The patient showed clinical improvement following compliance with the antitubercular drugs. No other major surgical or medical management-related complications were observed.

## Discussion

Little is known about the natural history of PA, considering its uncommon nature. The incidence is estimated to be approximately 0.4 cases per 100,000 population [6]. However, the prevalence is unknown, probably because of the lack of diagnostic testing data among asymptomatic patients. The caseload of PA has increased with a higher number of cases being detected with advancements in diagnostic modalities which can detect early changes before clinical signs are prominent [7]. The routine work in PA includes full blood count, CRP, and ESR, which suggest the ongoing inflammatory process [8].

Further investigations include blood cultures, USG, CT, and MRI. Imaging is done for confirming the diagnosis and planning the management. USG or CT-guided aspiration of the collection helps detect the causative organism through microscopy and cultures. These investigations also aid in the therapeutic drainage of larger collections [9]. Most importantly, they help in surgical planning by delineating the zone of involvement [10]. MRI helps understand the inflammatory extent of the lesion, which may involve adjacent structures including bone, thus helping in disease management. In our center, USG was the investigation of choice for diagnosis confirmation. MRI was not routinely ordered in our series. It was ordered only when patients had clinical signs of spinal involvement. CT is usually indicated to rule out any abdominal pathology (diverticulitis, abdominal wall abscess), other causes of retroperitoneal abscess, any visceral pathology, and the relations of abscess adjoining structures, as well as to detect any rupture into the peritoneal cavity. However, in our series, CT was performed mainly for surgical planning in patients with failed percutaneous therapeutic drainage. These observations suggest a mild-to-moderate illness without any systemic involvement.

India is a tuberculosis-endemic country. We administered anti-tubercular treatment to all patients considering the clinical features of a mild-to-moderate illness and cold abscess formation. No patient presented with severe infection features, including high-grade fever or systemic derangements. The possible reason for positive bacterial cultures could be from superadded infection and potentially from adjacent visceral infections. Additional culture-specific antibiotics took care of the non-tubercular infections. *Staphylococcus aureus* has been emerging as a major causative organism of PA [1,11-13]. The lower incidence of tuberculosis in the western population could be one factor. In our series, approximately 18% of cases had no organism detected in cultures, and another 27% had abscess volume too small and deep to be amenable for aspiration. In these cases, the anti-tubercular treatment was administered based on the clinicoradiological findings suggestive of tuberculosis and associated cold abscess.

Literature suggests a mortality of 2.4% in primary PA and 18.9% in secondary PA [14]. Death usually results from inadequate or delayed management when persistent collection remains the infective focus. The nil mortality in our series could be due to the less virulent nature of tuberculosis and because of timely drainage of the abscess and medical management. We did not encounter any patients with sepsis and severe infection-related symptoms, which could be another factor for low morality.

Undrained PA can result in higher mortality due to the persistence of infective focus [7]. Less invasive measures, such as percutaneous drainage, are superior with the obvious advantage of less pain, daycare treatment, and no need for anesthesia. Around half of our cases underwent USG-guided drainage through a pigtail catheter and one-fourth required open surgical drainage. Our experience supports the findings of Martins et al. [15], suggesting that percutaneous drainage is a minimally invasive, efficient, and safe procedure, with a good recovery and lower cost. Literature suggests that open drainage is usually required when concomitant abdominal pathology requires an open surgical procedure [16]. In our series, none of the patients had any concomitant visceral pathology requiring an open procedure. Failed percutaneous drainage and large abscess volume were the predictors of an open procedure in our series.

There are some limitations to this study. First, several aspects of PA management have not been adequately answered in the literature, which has resulted in treatment based on treating surgeons’ preferences in most cases. Hence, a uniform treatment protocol is difficult to formulate through the observations. Second, the study is based on the experiences of a North Indian population, which has shown different infective etiology patterns compared to the western population. Consequently, the results can have regional variation. Third, in the majority of the cases, the causative organism could not be isolated, and anti-tubercular treatment was initiated based on the clinicoradiological findings. Lastly, the findings are based on a small sample size due to an uncommon occurrence of this disease, and more long-term evidence is required to establish sound recommendations. Nevertheless, the study focuses on a less researched topic area, and the experience shared can be helpful to surgeons in tailoring their management and prognosticating patients with PA. Another important finding highlighted is that tuberculosis is the most common cause of PA in the Indian population. Finally, patients recover with anti-tubercular management even when no tissue/culture-based diagnosis is obtained.

## Conclusions

Tuberculosis is the most prevalent cause of PA in North India. Most patients respond well to the less invasive treatment with percutaneous therapeutic drainage and anti-tubercular drugs, with few patients requiring open drainage. However, tissue diagnosis may remain inconclusive in a few patients, and antitubercular treatment may need to be initiated based on the clinicoradiological evaluation. Nevertheless, the rate of complications is low, with nil mortality probably related to the mild-to-moderate disease course of tuberculosis.

## References

[1] Alonso CD, Barclay S, Tao X, Auwaerter PG (2011). Increasing incidence of iliopsoas abscesses with MRSA as a predominant pathogen. J Infect.

[2] Mallick IH, Thoufeeq MH, Rajendran TP (2004). Iliopsoas abscesses. Postgrad Med J.

[3] Ouellette L, Hamati M, Flannigan M, Singh M, Bush C, Jones J (2019). Epidemiology of and risk factors for iliopsoas abscess in a large community-based study. Am J Emerg Med.

[4] Mynter H (1881). Acute psoitis. J Buffalo Med Surg.

[5] Xu BY, Vasanwala FF, Low SG (2019). A case report of an atypical presentation of pyogenic iliopsoas abscess. BMC Infect Dis.

[6] Bartolo DC, Ebbs SR, Cooper MJ (1987). Psoas abscess in Bristol: a 10-year review. Int J Colorectal Dis.

[7] Ricci MA, Rose FB, Meyer KK (1986). Pyogenic psoas abscess: worldwide variations in etiology. World J Surg.

[8] Shields D, Robinson P, Crowley TP (2012). Iliopsoas abscess--a review and update on the literature. Int J Surg.

[9] Garner JP, Meiring PD, Ravi K, Gupta R (2007). Psoas abscess - not as rare as we think?. Colorectal Dis.

[10] Wall SD, Fisher MR, Amparo EG, Hricak H, Higgins CB (1985). Magnetic resonance imaging in the evaluation of abscesses. AJR Am J Roentgenol.

[11] López VN, Ramos JM, Meseguer V (2009). Microbiology and outcome of iliopsoas abscess in 124 patients. Medicine (Baltimore).

[12] van den Berge M, de Marie S, Kuipers T, Jansz AR, Bravenboer B (2005). Psoas abscess: report of a series and review of the literature. Neth J Med.

[13] Riyad MN, Sallam MA, Nur A (2003). Pyogenic psoas abscess: discussion of its epidemiology, etiology, bacteriology, diagnosis, treatment and prognosis-case report. Kuwait Med J.

[14] Gruenwald I, Abrahamson J, Cohen O (1992). Psoas abscess: case report and review of the literature. J Urol.

[15] Martins DL, Cavalcante Junior FA, Falsarella PM, Rahal Junior A, Garcia RG (2018). Percutaneous drainage of iliopsoas abscess: an effective option in cases not suitable for surgery. Einstein (Sao Paulo).

[16] Yacoub WN, Sohn HJ, Chan S (2008). Psoas abscess rarely requires surgical intervention. Am J Surg.

